# Corpus Spongiosum Abscess in a Patient Undergoing Intermittent Self-Dilatation: A Rare Case Report

**DOI:** 10.7759/cureus.69382

**Published:** 2024-09-14

**Authors:** Akshay Vinoo, Meyada Ali, Naveen Sivakumar, Anil Krishan, Abida Sultana

**Affiliations:** 1 Urology, Worcestershire Acute Hospitals NHS Trust, Worcester, GBR; 2 General Surgery, Worcestershire Acute Hospitals NHS Trust, Worcester, GBR; 3 Urology, Sandwell and West Birmingham NHS Trust, Birmingham, GBR; 4 General Surgery, University Hospitals Birmingham, Birmingham, GBR; 5 Urology, Charing Cross Hospital, London, GBR

**Keywords:** abscess, andrology, corpus spongiosum, intermittent self dilatation, urology

## Abstract

Penile abscesses of the corpus spongiosum are rare in urology, with few documented cases. These abscesses may occur spontaneously or due to risk factors such as diabetes mellitus, intracavernosal injection therapy, tuberculosis, trauma, and perianal or intra-abdominal abscesses. This report discusses a 76-year-old man who developed a penile abscess involving the corpus spongiosum following intermittent self-dilatation. This required open drainage together with antibiotic cover to clear the infection, and follow-up in an andrology clinic found no remnant abscess. This case highlights the importance of early diagnosis and intervention in penile abscesses, typically managed with imaging, drainage, and culture-directed antibiotics. The drainage options may include open or an ultrasound-guided approach, depending on the size and location. A multidisciplinary approach is crucial, with careful follow-up to manage potential complications such as penile deviation and erectile dysfunction. Pre-procedural counseling is essential, particularly in cases involving urethral instrumentation.

## Introduction

Penile abscesses in urology are uncommon, with limited cases reported in the literature. Approximately one-third occur spontaneously [1]; however, risk factors include diabetes mellitus, intracavernosal injection therapy, tuberculosis, trauma (including foreign bodies), priapism, perianal, perineal, and intra-abdominal abscesses, as well as pre-existing pathology such as urethral diverticulum [1-7]. The most common causative organisms include *Staphylococcus aureus*, *Streptococci*, *Bacteroides*, and *Enterococci*. Further cases of infection with* Escherichia coli* and *Mycobacterium tuberculosis* have been described [1,3]. Treatment modalities are varied, ranging from ultrasound-guided drainage to open drainage, depending on the anatomical location and size of the collection [1-12].

Most reported penile abscesses are cavernosal; the corpus spongiosum is rarely affected [8-12]. We report an unusual case of a 76-year-old man developing a penile abscess involving the corpus cavernosum and spongiosum at presentation, following previous urological instrumentation.

## Case presentation

A 76-year-old gentleman with a background of hypertension, benign prostatic hyperplasia, and previously treated nasal carcinoma initially presented to the Urology Department via the general practitioner with visible hematuria. He subsequently underwent a flexible cystoscopy, which showed bladder debris with poor views and a possible bladder stone. Urine microscopy and culture were sent off during this visit. The following month, he underwent rigid cystoscopy and was found to have a proximal bulbar stricture. This stricture was not reported during the previous flexible cystoscopy as the flexible cystoscope had a caliber of 16 Fr that was able to navigate through the stricture. The bulbar stricture was dilated to 24 Fr using serial S-shape dilators over a guide wire under suitable antibiotic cover on anesthetic induction as per urine cultures. On examination of the bladder, debris was found and washed out. There was no evidence of a stone. He was discharged with a plan to a trial without catheter (TWOC) in two weeks. 

During the TWOC visit, he was found to have a large post-void residual and was advised to perform intermittent self-catheterization (ISC) after being demonstrated and taught how to do this safely. This was done for a year until he presented acutely with urinary retention, which required the insertion of a 16 Fr urinary catheter. He was asked to return in two weeks for a TWOC.

During his subsequent TWOC visit, the patient reported swelling at the base of his penis, which he had noticed for the past three months. This had evolved into per urethral discharge over the past three days. He did not complain of any systemic symptoms, such as vomiting, fever, or history of weight loss. He was afebrile and hemodynamically stable on admission. 

On examination, the catheter was in situ and draining clear urine. However, there was green, malodorous purulent discharge from the meatus. On palpation, there was a tender, firm lump present at the base of the penis, with pus discharging from the meatus on pressure over the lump. Further examination findings included scrotal erythema with intact skin and no necrotic patches visible.

Blood tests on admission showed raised inflammatory markers, with a CRP of 183 mg/L (normal range: 1-5 mg/L) and a white cell count of 14 x 10^9^/L (normal range: 4-11 x 10^9^/L) with predominant neutrophilia.

**Figure 1:**
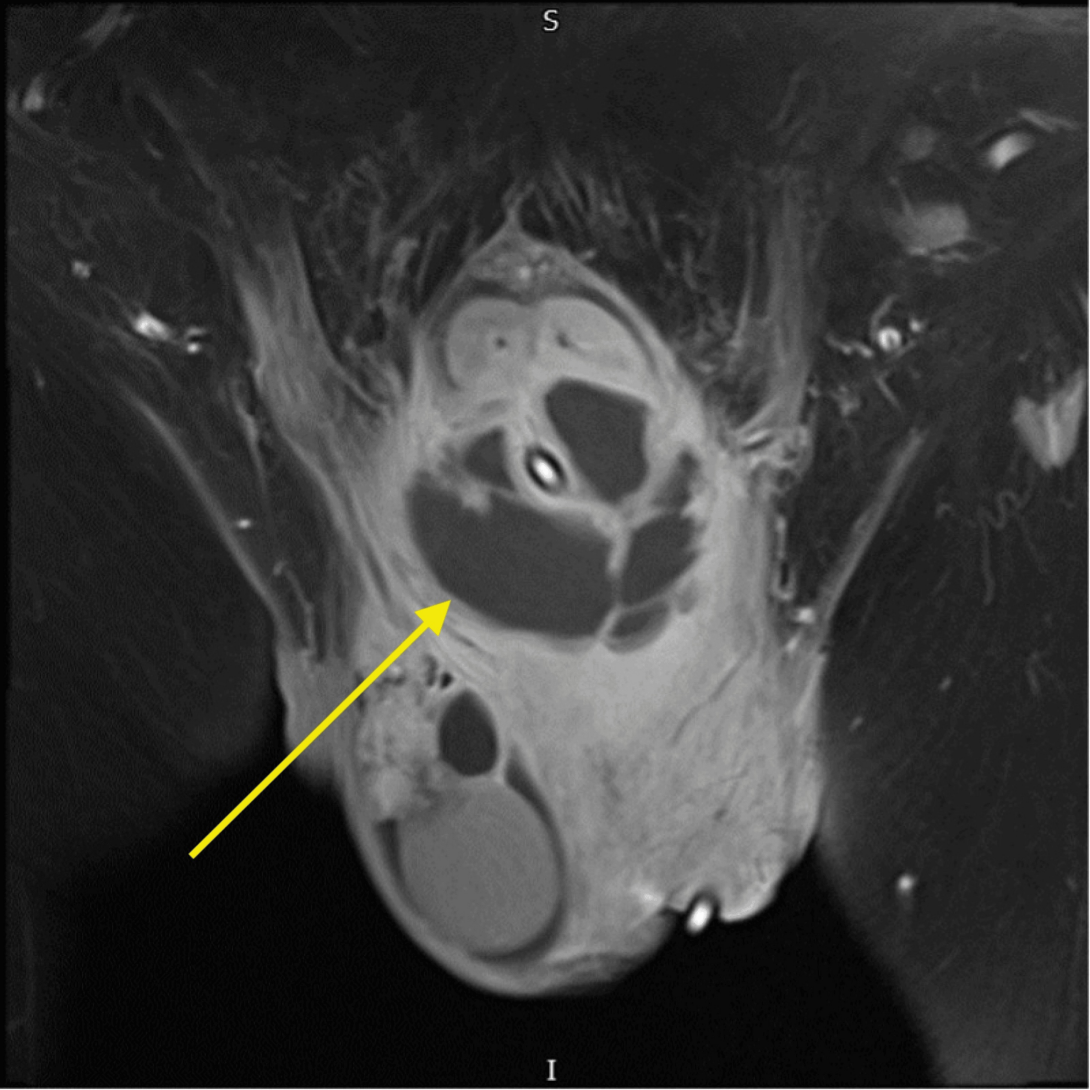
MRI T1 coronal image of the perineum. Yellow arrow highlighting the area of low attenuation, suggestive of an abscess.

This patient was managed on admission with intravenous antibiotic treatment (gentamicin 5 mg/kg actual body weight IV stat + ciproflocacin 400 mg IV TDS for one day, then switched to tazocin 4.5 g IV TDS for the next four days). An MRI of the pelvis performed subsequently showed a 70 x 46 mm peripherally enhancing fluid collection involving both the corpora cavernosa on either side and the corpus spongiosum, most likely in keeping with an abscess. An MRI cross-sectional image of the collection is shown in Figure 1. The following day, this abscess spontaneously ruptured, and a urinary catheter was inserted, which drained clear urine. A urethral wound swab done at the time grew *Streptococcus anginosus,* which was sensitive to penicillin. The urine culture done at the time did not show any growth. He was discharged home with oral antibiotics and referred urgently to the regional andrology center.

He underwent an incision and drainage of the abscess at the regional center six days after his original presentation. An elliptical incision of the mid penile shaft revealed a 1 cm defect in the urethra on the dorsal aspect, and pus was evacuated. Histology confirmed active inflammation and granulation tissue in keeping with an abscess, with no evidence of malignancy. He was discharged with oral antibiotics and catheter in situ.

He was reviewed in an andrology clinic after four months, where no clinically palpable abscess was found. He underwent a flexible cystoscopy a month later, and he was found to have a short segment bulbar urethral stricture. The patient, who had trouble with voiding lower urinary tract symptoms before the penile abscess, found having the urinary catheter significantly improve his quality of life. Hence, a new catheter was replaced during the same flexible cystoscopy session.

## Discussion

The penis is made up of two cavernous bodies (corpora cavernous) and a single corpus spongiosum ventromedially surrounded by the tunica albuginea. The corpora cavernosa is surrounded by Buck's fascia, which separates it from the corpus spongiosum [8]. Abscesses are most commonly found in the corpus cavernosa [1]. They rarely involve the spongiosum; in this case, the abscess extended to involve both corpora cavernosa and the spongiosum. 

The most common presenting complaints of penile abscess include a painful penile mass, swelling, and urethral discharge. Diagnosis can be confirmed with imaging, including US, CT, or MRI [1,6-8]. Ultrasound-guided drainage can occur concomitantly [8,10]. Further investigations such as urine, blood, and pus culture will guide the duration and type of antimicrobial treatment.

Treatment options for cavernosal abscesses include antibiotic cover as per sensitivities available, USG-guided drainage, and open drainage. Open drainage is the most favored of all modalities for drainage [1-3,5-7,9,11,12]. Variability in management is prevalent. In cases with a well-defined abscess, USG-guided drainage has proven successful with appropriate antibiotic cover [4,8,10]. Isolated cases had varied management plans depending on the presentation. Dugdale et al. reported a case of an idiopathic corpus cavernosum abscess managed with exploration, which was complicated by a cutaneous fistula requiring a protracted course of antibiotics [1]. In one particular case, a recurrent cavernosal abscess secondary to MRSA (methicillin-resistant *Staphylococcus aureus*) required a total penectomy with perineal urostomy for infection clearance [6]. Open drainage with antibiotic cover was favored in another case as part of an extension of an intra-abdominal abscess [5].

USG-guided drainage was used in a corpus spongiosum abscess formed as a result of an *Enterococcus* urinary tract infection (UTI) and in that of a spontaneous abscess formed in the case of a diabetic patient. In the latter, antifungal cover was required as *Candida* was grown in cultures [8,9]. Due to the close proximity of organs in the perineum, extension from adjacent structures can predispose to corpus spongiosum abscesses. This reflects the use of open drainage to manage two cases of abscesses formed: one due to rectal cancer invasion and the other due to a complicated fistula in ano [10,11]. In a case similar to ours, urethral dilatation with a balloon dilator was caused an abscess that required open drainage. In our case, serial S-shape dilators were used, which, however, did not change the treatment modality used [12].

In general, the management of penile abscesses follows the principles similar to other abscesses: early identification with appropriate imaging, early exploration or drainage as per the nature of the collection, and culture-directed antibiotics. A multi-disciplinary team approach is important. Daily reviews and close discussion with microbiology will dictate further re-explorations and the duration of antibiotic course. The follow-up of these patients remains unclear considering the varied presentations and management options. Penile deviation and erectile dysfunction on follow-up have been well reported in the literature, and early counseling may help manage expectations. Additionally, as seen in our case, the iatrogenic nature of the injury makes it important to explain this risk during the consenting process for urethral dilatation and the counseling process for ISC.

## Conclusions

Penile abscesses and, in particular, corpus spongiosum abscesses are a rare entity. We highlight a case in which ISC has potentially led to an abscess. This could have been caused by the trauma from the ISC, inappropriate technique, or a combination of both in the presence of a UTI. Awareness of this is necessary to counsel patients appropriately and ensure that a safe technique is practiced after adequate supervised ISC training. The management of such abscesses depends on the site, size, and associated morbidity, with options ranging from antimicrobial cover to drainage via open or ultrasound guidance. Andrology follow-up in a timely manner would help identify potential complications such as erectile dysfunction and penile deviation, which may need subsequent management. The duration of follow-up will need to be dictated by the local availability of resources and the functional expectations of the patient.
